# Real-life cost-effectiveness of benralizumab in patients with severe asthma

**DOI:** 10.1186/s12931-021-01758-0

**Published:** 2021-05-27

**Authors:** A. Padilla-Galo, A. J. García-Ruiz, R. Ch. Levy Abitbol, C. Olveira, F. Rivas-Ruiz, N. García-Agua Soler, M. Pérez Morales, B. Valencia Azcona, B. Tortajada-Goitia, I. Moya-Carmona, A. Levy-Naon

**Affiliations:** 1 Pneumology Unit, 4th Floor, Agencia Sanitaria Costa del Sol, Carretera Nacional 340, Km 187, 29603 Marbella, Málaga, Spain; 2 Chair of Health Economics and Rational Use of Medicines, Department of Pharmacology and Clinical Therapeutics, Faculty of Medicine, University of Málaga, Málaga, Spain; 3 Yeshiva University, New York, USA; 4 Pneumology Department, IBIMA (Institute for Biomedical Research of Málaga), Regional University Hospital of Málaga, Avenida Carlos Haya, 29010 Málaga, Spain; 5 University of Málaga, Málaga, Spain; 6 Research Unit, Red de Investigación en Servicios de Salud en Enfermedades Crónicas, REDISSEC (Spanish Healthcare Network for Chronic Diseases), Agencia Sanitaria Costa del Sol, Carretera Nacional 340, Km 187, 29603 Marbella, Málaga, Spain; 7 Pharmacy and Nutrition Service, Agencia Sanitaria Costa del Sol, Carretera Nacional 340, Km 187, 29603 Marbella, Málaga, Spain; 8 Pharmacy and Nutrition Service, Hospital Universitario Virgen de La Victoria, Campus de Teatinos s/n, 29010 Málaga, Spain; 9 Pneumology Department, Hospital Universitario Virgen de La Victoria, Campus de Teatinos s/n, 29010 Málaga, Spain

**Keywords:** Asthma, Benralizumab, Cost-effectiveness, Economic impact, Eosinophils, Biologics, Severe asthma, Real-life

## Abstract

**Background:**

Availability of clinically effective and cost-effective treatments for severe asthma would be beneficial to patients and national healthcare systems. The aim of this study was to evaluate clinical outcomes and healthcare expenditure after incorporating benralizumab into the standard treatment of refractory eosinophilic asthma.

**Methods:**

This was a cross-sectional multicentre study of consecutive patients with refractory eosinophilic asthma who received treatment with benralizumab during at least 12 months. Patient follow-up was performed in specialised severe asthma units. The main effectiveness parameters measured were: the avoidance of one asthma exacerbation, a 3-point increase in the asthma control test (ACT) score, and the difference in utility scores (health-related quality of life) between a 1-year baseline treatment and 1-year benralizumab treatment. The health economic evaluation included direct costs and incremental cost-effectiveness ratios (ICERs).

**Results:**

After 1 year of treatment with benralizumab, patients with refractory eosinophilic asthma showed an improvement in all the effectiveness parameters analysed: improvement of asthma control and lung function, and decrease in the number of exacerbations, oral corticosteroid (both as corticosteroid courses and maintenance therapy), and inhaled corticosteroid use. The total annual cost per patient for the baseline and benralizumab treatment periods were €11,544 and €14,043, respectively, reflecting an increase in costs due to the price of the biological agent but a decrease in costs for the remaining parameters. The ICER was €602 per avoided exacerbation and €983.86 for every 3-point increase in the ACT score.

**Conclusions:**

All the pharmacoeconomic parameters analysed show that treatment with benralizumab is a cost-effective option as an add-on therapy in patients with refractory eosinophilic asthma.

## Background

Asthma is a heterogeneous condition characterised by chronic inflammation of the pulmonary airways [1] with an estimated 300 million people currently affected worldwide [2], and its prevalence has been increasing in recent years. Severe asthma is defined as asthma that requires maximal, optimised inhaled corticosteroid (ICS) therapy plus another controller treatment to remain under control, or asthma that is uncontrolled despite this therapy [1, 3]. It is estimated to affect 5–10% of the asthma population. Refractory eosinophilic asthma is a phenotype of severe asthma characterised primarily by increased blood eosinophils and frequent exacerbations despite corticosteroid therapy [1, 3]. Several studies have shown that despite the availability of effective treatments such as ICS, long-acting β2-agonists (LABA), tiotropium, and leukotriene modifiers, over 50% of asthma patients are assessed as not well-controlled in standard clinical practice [4–6] and many require further therapies such as oral corticosteroids (OCS) and biologics [7]. Over 50% of deaths caused by asthma are reported in patients with a history of severe asthma [8] and this severe condition is associated with increased morbidity, healthcare costs, and mortality [9, 10].

Availability of clinically effective and cost-effective treatments for severe asthma would be beneficial to patients and national healthcare systems. The use of biological therapies could lead to improved clinical outcomes in severe asthma together with a reduced economic burden of the disease. There are currently four biologic agents available in Spain for the treatment of severe asthma: omalizumab, mepolizumab, reslizumab, and benralizumab. Several studies performed in different healthcare settings have provided evidence on the advantages of omalizumab [11–13], mepolizumab [14, 15], and reslizumab [16] as add-on therapies in terms of cost-effectiveness in the management of severe asthma patients.

Benralizumab (Fasenra^®^, AstraZeneca) binds to the human IL-5 receptor (IL-5R) through its Fab domain, thereby preventing IL-5 from binding to its receptor and inhibiting differentiation and maturation of eosinophils in the bone marrow. In addition, this antibody has the ability to bind through its afucosylated Fc domain to the RIIIa region of the Fc receptor on natural killer cells, macrophages, and neutrophils, thereby enhancing antibody-dependent cell-mediated cytotoxicity of both blood eosinophils and tissue-resident eosinophils [17, 18]. In three phase 3 clinical trials (SIROCCO [19], CALIMA [20], and ZONDA [21]), the administration of 30 mg subcutaneous benralizumab every 8 weeks (every 4 weeks for the first three doses) reduced the annual rate of severe asthma exacerbations and the use of OCS, and improved symptom control and lung function determined by the forced expiratory volume in 1 s (FEV_1_). Additionally, the BORA study [22] has shown its long-term efficacy and safety. Despite having been approved only recently, other studies are confirming these good results also in real life [23–25]. To date, however, no real-life pharmacoeconomics studies of benralizumab have been conducted. The purpose of this study was therefore to assess the cost-effectiveness of benralizumab therapy and its 1-year effectiveness, based on the decrease in the number of exacerbations and OCS use, and the improvement of asthma control and lung function in the real world.

## Methods

### Study population and study design

This multicentre study included 44 patients with refractory eosinophilic asthma who received treatment with benralizumab for at least 12 months at the Asthma Units of Hospital Costa del Sol (Marbella, Spain) and Hospital Virgen de la Victoria (Málaga, Spain). All patients were diagnosed with asthma based on objective tests (FEV_1_ reversibility ≥ 12%, positive results to methacholine, or FEV_1_ variability ≥ 20%).

A standardised protocol was used to try to improve these patients’ asthma control. This consisted of ensuring adherence to therapy and appropriate inhaler use, providing health education, adjusting treatment, and ruling out comorbidities [26–28].

Benralizumab treatment initiation criteria were as follows:

- 18-year-old patient or older with refractory eosinophilic asthma [3];
- GINA guidelines step 5 [1];
- 2 or more exacerbations during the previous year with use of OCS despite receiving appropriate treatment for the degree of severity or corticosteroid dependence;
- Presence of eosinophilic inflammation: eosinophil count ≥ 300 cells/µL in peripheral blood during the previous 12 months or ≥ 150 cells/µL in case of corticosteroid dependence.

All patients were treated with benralizumab for at least 12 months and were included in the analysis.

Patients previously treated with another biologic agent but who had failed to respond based on the physician’s judgement were included. The following criteria for lack of response to a prior biologic treatment were applied:

- Continued use of maintenance OCS despite receiving biologic therapy for at least 12 months, or
- Less than a 50% reduction in exacerbations after at least 12 months of biologic therapy.

At least three visits were performed after treatment initiation with benralizumab: one at 3 months of treatment, one at 6 months of treatment, and a final visit at 12 months of benralizumab treatment.

Written informed consent was obtained from all participants. The study was reviewed by the Spanish Medicines and Health Products Agency and approved by the ethics committee *Comité de ética provincial de Málaga*.

### Clinical, analytical, and lung function variables

A database was compiled from complete medical records, with data from diagnosis to study enrolment. A standardised protocol was applied for the prospective collection of sociodemographic data (sex, age), clinical profile (age at diagnosis of asthma, smoking, atopy, presence of nasal polyps), exacerbations, use of corticosteroid therapy, and basic blood test. Dyspnoea was evaluated by means of the modified Medical Research Council Scale for Dyspnoea [29], and we divided patients into two stage groups (0–2 and 3–4) according to their degree of dyspnoea. We used the asthma control test (ACT) to evaluate the degree of asthma control in the 4 weeks prior to the clinical interview. The ACT [30] is a self-administered tool that is easy for patients to complete. It includes four symptom-relief questions plus a patient’s self-assessment of asthma control [1] in the last 4 weeks, with scores ranging from 5 (poor control) to 25 (complete control), and has been validated in Spanish [31]. A 3-point difference in the ACT score has been estimated as a minimally important difference [32]. Nasal polyposis, a frequent comorbidity in severe asthma, was diagnosed by an otorhinolaryngologist by direct visualisation of the polyps with endoscopic examination. Patients were considered as atopic when they had positive allergic prick tests or positive specific IgE to the most prevalent pneumo-allergens in our area, provided that these positive findings also had clinical relevance. Corticosteroid dependence was defined as the daily use of OCS during at least 6 months. Lastly, severe asthma exacerbations, defined as exacerbations requiring the use of systemic corticosteroids for at least 3 days and/or leading to an emergency department visit and/or hospital admission, were studied [33].

All patients were trained to identify exacerbation symptoms. They were also asked to record detailed information about their condition and their prescriptions (systemic steroids). This information was verified in their medical records.

Fractional exhaled nitric oxide (FeNO) was measured with a conventional chemiluminescence analyser (NIOX, Aerocrine AB, Sweden) using the online standardised single-breath technique, and was followed by the performance of a spirometry. Both procedures conformed to international guidelines [34, 35].

Patients were classified according to their response at 12 months of benralizumab treatment as patients with complete response, patients with controlled asthma, patients with partial response, and patients with no response, based on the Spanish consensus for severe asthma in adults [36]. Response criteria are shown in Table 1.

**Table 1:** Classification based on the response to a biologic treatment for severe asthma. Reproduced from [36]

	Exacerbations^a^	ACT	FEV_1_	Systemic corticosteroids
No response	Identical or increased number	< 3-point increase	< 10% and 100 mL increase	< 50% decrease
Partial response	< 50% reduction ≥ 2 severe exacerbations in 12 months	< 3-point increase Total score < 20	> 10% and 100 mL increase FEV_1_ < 80%	> 50% dose decrease No OCS discontinuation
Controlled asthma	≤ 1 severe exacerbation in 12 months	Total score ≥ 20	FEV_1_ < 80%	OCS discontinuation
Complete response	No exacerbations in 12 months	Total score ≥ 20	FEV_1_ ≥ 80%	OCS discontinuation

^a^Taking into account the number of exacerbations during the preceding year

All variables were measured during the baseline visit and at 3, 6 and 12 months of treatment.

### Outcomes

Three different measures of effectiveness were considered for the pharmacoeconomics analysis:

1. Avoidance of one asthma exacerbation [33],
2. 3-point increase in the ACT score [32],
3. Difference in utility (health-related quality of life) between baseline treatment and benralizumab treatment (EQ-5D index obtained from Spanish asthmatic patients [37]). To calculate utility values, we used the benefits obtained in Spain according to the ACT score in patients with severe asthma [37]. Utility values were as follows: 0.91 for patients with controlled asthma and 0.73 for patients with uncontrolled asthma.

Other parameters of effectiveness such as reduction in severe exacerbations and emergency department visits, reduction in the use of oral and inhaled steroids, and improvement of lung function and asthma control based on the ACT score were measured.

### Cost analysis

Direct healthcare costs of these patients’ management during the year prior to the start of benralizumab were determined and compared with the direct healthcare costs at 1 year on benralizumab treatment. Direct costs included pharmacological costs and use of healthcare resources. Pharmacological costs included medications for the management of severe asthma, such as ICS, OCS, and monoclonal antibodies (omalizumab, mepolizumab, reslizumab, and benralizumab). Healthcare resources considered were: tests (spirometry, bronchodilator test, FeNO, skin prick, haemogram, biochemistry, and chest CT) carried out at visits to the asthma unit during the year prior to the start of benralizumab treatment and the year on benralizumab treatment, hospital admissions due to exacerbations, recorded number of emergency department visits (primary care emergency visits and hospital emergency visits), and prednisone courses administered during exacerbations.

The costs of the healthcare resources used were obtained from the Andalusian Healthcare Service [38] and the costs of the medicines used were obtained from the Spanish Ministry of Health and from the Summary of Product Characteristics of each product [39, 40]. Costs were determined and expressed in 2020 Euros (€) (Table 2).

**Table 2:** Costs of healthcare resources used [38–40]

Healthcare resource used	Unit cost in €
Emergency visit	144.24
Emergency visit + observation	392.03
Hospital stay/day	495.59
Hospital stay pneumology/day	386.65
Spirometry	40.57
Spirometry + bronchodilation	60.49
Ig E determination	21.50
FeNO determination	39.00
Prick test	134.70
Haemogram	5.30
Biochemistry	83.50
Non-contrast CT	55.38

Drug	Dose	Therapeutic regimen	Annual cost
Omalizumab	150 mg	Based on both IgE level and weight	17,042.61
Mepolizumab	100 mg	100 mg/4 weeks	10,661.43
Benralizumab	30 mg	Dose 30 mg/4 weeks; 3 doses; then 30 mg/8 weeks	13,762.64
Corticosteroids			
Oral	5 mg	5 mg/day; 7-day course	11.86
	30 mg	30 mg/day; 7-day course	23.73
Inhaled	200 µg	Pack size: 200 puffs	36.96

### Pharmacoeconomics analysis

Cost-effectiveness analyses (CEAs)—defined in a broad sense, i.e., including cost utility analyses (CUAs)—are faced with the difficulty of having to use different units for measuring costs and health outcomes, as costs are expressed in monetary units and health outcomes are measured in physical/clinical units or quality-adjusted life-years (QALYs). Therefore, a programme cannot be accepted or rejected in absolute terms, but only in relation to another programme which acts as a term of comparison, reference, or control.

**Fig. 1:**
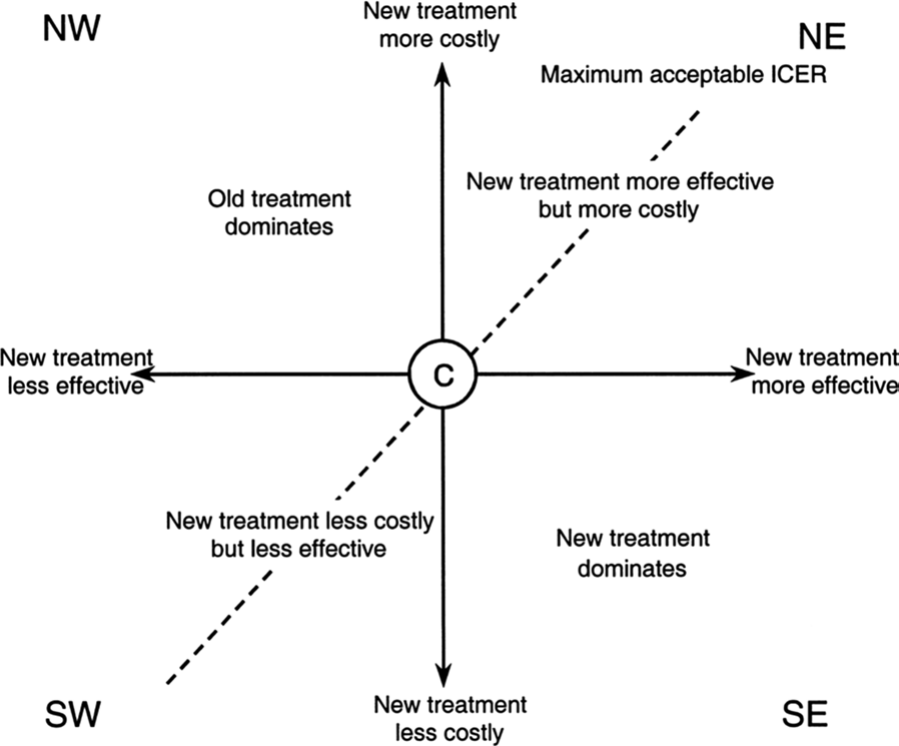
Decision rules for cost effectiveness analysis

All possible cost-effectiveness comparisons can be represented graphically on the so-called cost-effectiveness plane or space (Fig. 1), with differences in effectiveness plotted along the X-axis and differences in costs plotted along the Y-axis. Thus, the quadrants of the cost-effectiveness plane indicate four possible situations. The figure clearly illustrates that decision rules are required only for the cost-effectiveness pairs situated in the NE and SW quadrants.

Therefore, decision rules in CEAs -and CUAs- are based on the comparison of cost increases (∆C) with effectiveness increases (∆E) to calculate the extra cost per additional unit of effectiveness of the programme being evaluated in relation to the reference programme. The incremental cost-effectiveness ratio (ICER) was used to compare the impact of benralizumab treatment in terms of costs and clinical outcomes with the baseline treatment both over a 1-year period, and was calculated as follows:

$${\text{ICER}} = \frac{{{\text{Cost}}_{{{\text{post}}}} - {\text{Cost}}_{{{\text{pre}}}} }}{{{\text{Effectiveness}}_{{{\text{post}}}} - {\text{Effectiveness}}_{{{\text{pre}}}} }}$$

### Sensitivity analysis

A probabilistic sensitivity analysis was carried out using a first-order Monte Carlo simulation in a hypothetical cohort of 10,000 patients for both costs and effectiveness for the two regimens under study: baseline regimen (pre-benralizumab) versus new regimen (benralizumab).

Our pharmacoeconomics analysis also took into account the net benefit obtained both in monetary and nonmonetary (health outcomes) terms.

The Net Monetary Benefit (NMB) is a summary statistic that represents the value of an intervention in monetary terms when the willingness to pay (WTP) threshold for a benefit unit (e.g., a health outcome measure or QALY) is known. The NMB is calculated as follows:

$${\text{Net monetary benefit}} = \left( {{\text{E}}*{\text{WTP}}} \right){-}{\text{C}}$$

where E = effectiveness; WTP = willingness-to-pay threshold; C = cost.

In Spain, the WTP threshold varies between €22,000 and €24,000 per year [41].

The Net Health Benefit (NHB) was also used. This is a summary statistic representing the impact of a new intervention on the health of the population. The NHB assumes that “lost health” can be estimated as an “opportunity cost” to represent health lost as a result of the displacement of resources to fund a new intervention. NHB is normally measured using QALYs and calculated as: incremental benefit − (incremental cost/WTP threshold). Given a certain WTP, a positive NHB implies that the general health of the population would increase as a result of the new intervention, while a negative NHB implies that the health benefits of the new intervention are not sufficient to compensate for the health losses arising from health care that is no longer funded to finance the new treatment.

### Statistical analysis

All the data were analysed using the software SPSS v25 licensed from the University of Malaga. A descriptive analysis was performed using measures of central tendency, position, and dispersion for quantitative variables, and frequency distribution for qualitative variables. To assess changes at 3, 6, and 12 months compared with baseline, Student’s t test for paired samples was used (Wilcoxon rank test for non-normal distributions) and McNemar test was used for qualitative variables. A generalised linear model was used for the analysis of repeated measures with four assessment timepoints.

Effect-size calculations were performed to differentiate between statistically significant and clinically relevant results [41, 42]. By using Cohen’s “d” index we can determine the degree of association between two variables or their differences. Cohen’s “d” index allows to quantify the effect of treatments in relation to the clinical criterion analysed, and effect sizes can be classified as: insignificant effect (− 0.15 and < 0.15); small effect (≥ 0.15 and < 0.40); medium effect (≥ 0.40 and < 0.75); large effect (≥ 0.75 and < 1.10); very large effect (≥ 1.10 and < 1.45); enormous effect > 1.45 [43, 44].

Confidence intervals (CIs) for the ICERs were computed based on bootstrapping, using sampling with replacement (the size of the new samples was equal to the original size). We ran 10,000 Monte Carlo simulations, and the 2.5th and 97.5th percentiles from the simulations were used to determine a 95% CI. Statistical significance was set at p < 0.05.

## Results

### Patient population

A total of 44 patients with refractory eosinophilic asthma who received benralizumab treatment during at least 12 months were enrolled. Clinical characteristics of the study population are shown in Table 3. In short, most patients were women in their fifth decade of life, overweight, and with significant eosinophilic inflammation as evidenced by the presence of eosinophilia. In addition, all patients were receiving high dose ICS, LABA, and at least one other controller.

**Table 3:** Baseline patient characteristics

Parameter	n = 44
Age, years (mean ± SD)	53.8 ± 10.4
Women, n (%)	35 (79.5)
BMI (mean ± SD)	28.7 ± 6
Smoking	
Never smoker, n (%)	23 (52.3)
Ex-smoker, n (%)	21 (47.7)
Former smoker, n (%)	0 (0)
Age at diagnosis, years (mean ± SD)	28.95 ± 12.4
Dyspnoea	
Degree 0–2, n (%)	22 (50)
Degree 3–4, n (%)	22 (50)
Atopy, n (%)	15 (34.1)
Corticosteroid-dependent, n (%)	18 (40.9)
Nasal polyps, n (%)	14 (31.8)
AERD, n (%)	6 (13.6)
ACT (mean ± SD)	13.7 ± 4
ED visits in the previous year, (mean ± SD)	4.1 ± 2.6
Number of severe exacerbations in the previous year, (mean ± SD)	5.50 ± 2.63
Number of asthma admissions in the previous year, (mean ± SD)	0.59 (1.1)
Courses of OCS in the previous year, (mean ± SD)	5.8 ± 3.3
Oral prednisone (or equivalent) dose, mg/day (mean ± SD)	19.3 ± 8.8
Inhaled budesonide (or equivalent) dose, μg/day (mean ± SD)	993 ± 485
Post-BD FEV_1_, mL (mean ± SD)	1455.8 ± 495.7
Post-BD FEV_1_, % (mean ± SD)	65.7 ± 14.1
FeNO, ppb (mean ± SD)	56.6 ± 26.2
Blood eosinophil count, cells/μL (mean ± SD)	718.3 ± 287.5
IgE, IU/mL (mean ± SD)	223.6 ± 394.9
Prior treatment with a biologic agent, n (%)	23 (52.3)
Omalizumab, n (%)	16 (36.4)
Mepolizumab, n (%)	5 (11.4)
Omalizumab + Mepolizumab, n (%)	2 (4.5)
Time with previous biological treatment, months (mean ± SD)	20.7 ± 23.3

*ACT* asthma control test, *AERD* aspirin-exacerbated respiratory disease, *BD* bronchodilator, *BMI* body mass index, *ED* emergency department, *FeNO* fractional exhaled nitric oxide, *FEV*_*1*_ forced expiratory volume in 1 s, *ppb* parts per billion, *OCS* oral corticosteroids, *SD* standard deviation

### Parameters assessed

**Fig. 2:**
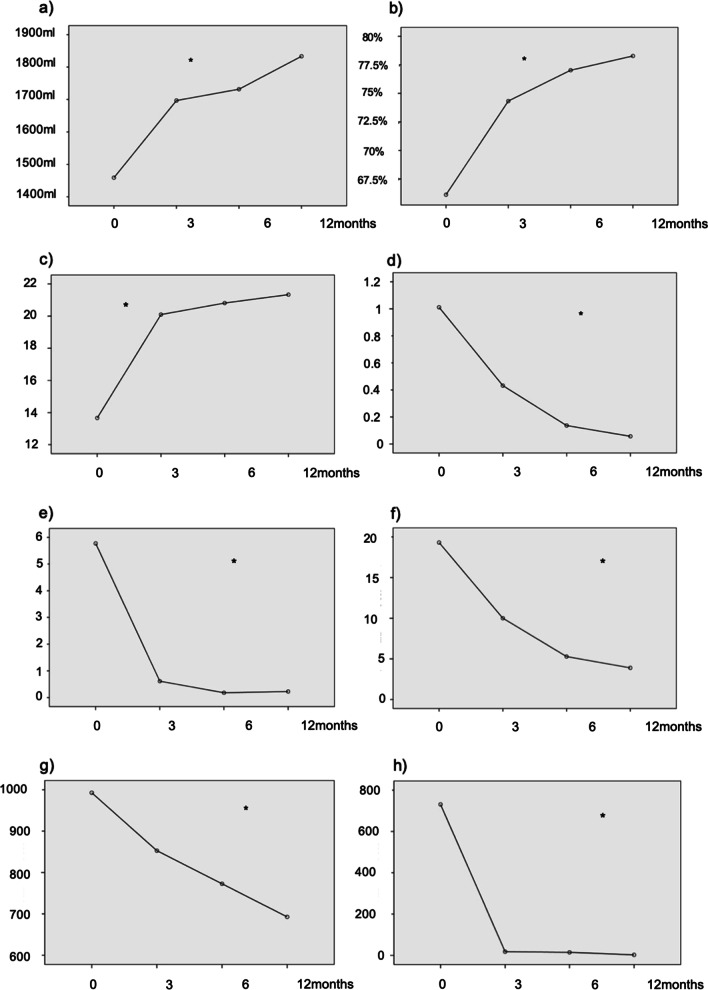
Clinical, functional, and laboratory data at baseline and at 3, 6, and 12 months of treatment. **a** FEV_1_ mL; **b** FEV_1_%; **c** ACT (asthma control test); **d** No. of emergency department visits: **e** No. of oral corticosteroid courses; **f** Oral prednisone dose (mg/day); **g** Inhaled budesonide dose (μg/day); **h** Blood eosinophils (cells/μL). Data expressed as means. *p < 0.001

Clinical, functional, and laboratory data at baseline and at 3, 6, and 12 months of treatment as well as the comparison between values at baseline and at 12 months are presented in Table 4 and Fig. 2.

**Table 4:** Clinical, functional, and laboratory data at baseline and at 3, 6, and 12 months of treatment

Variables	Baseline	3 months	6 months	12 months	p*
ACT, mean (SD)	13.7 (4.1)	20.1 (3.6)	20.8 (2.9)	21.3 (2.2)	**< 0.001**
Controlled asthma (ACT ≥ 20), n (%)	2 (4.5)	25 (56.8)	36 (81.8)	39 (92.9)	**< 0.001**
Number of ED visits in the previous year, mean (SD)	4.1 (2.6)	–	–	0.7 (1.6)	**< 0.001**
Number of severe exacerbations in the previous year, mean (SD)	5.50 ± 2.63	–	–	0.66 ± 0.94	**< 0.001**
Corticosteroid-dependent, n (%)	18 (40.9)	16 (36.4)	9 (20.5)	8 (18.2)	**< 0.001**
Inhaled budesonide (or equivalent) dose, μg/day, mean (SD)	993 (485)	853 (446)	773 (408)	693 (343)	**< 0.001**
Oral prednisone dose, mg/day, mean (SD)	19.3 (8.8)	10 (8.6)	5.3 (7.8)	3.9 (7.2)	**< 0.001**
Number of courses of OCS (previous year), mean (SD)	5.8 (3.3)	–	–	1 (1.6)	**< 0.001**
FEV_1_ mL, mean (SD)	1459 (509)	1697 (495)	1732 (621)	1833 (556)	**< 0.001**
FEV_1_%, mean (SD)	66.1 (14)	74.3 (12.2)	77 (14.5)	78.3 (15)	**< 0.001**
Blood eosinophil count, cells/μL, mean (SD)	730.9 (288)	18 (18)	14.9 (13.7)	2.7 (4)	**< 0.001**

*ACT* asthma control test, *ED* emergency department, *FEV*_*1*_ forced expiratory volume in 1 s, *OCS* oral corticosteroids, *SD* standard deviation

^*^Comparison between data at baseline and at 12 months

At 1 year of treatment, there was an 83% reduction in emergency department visits, an 88% reduction in severe exacerbations, a 79.8% reduction in the prednisone (or equivalent) dose, a 55.6% reduction in the number of corticosteroid-dependent patients, and an 82.8% reduction in the number of OCS courses. 65.9% of patients had required zero emergency department visits during the 1-year treatment with benralizumab and 47.7% consumed zero OCSs (both as corticosteroid courses and maintenance therapy) during that period.

**Fig. 3:**
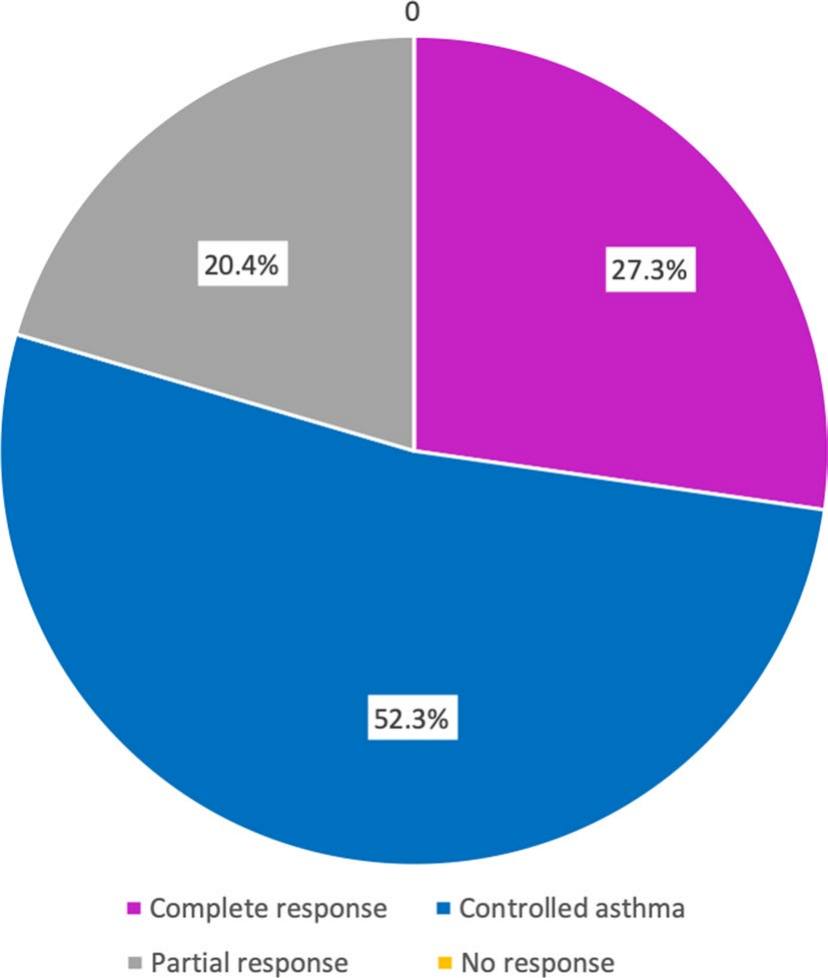
Classification based on response at one year of benralizumab treatment

We classified patients according to their response at 12 months of benralizumab treatment based on the Spanish Severe Asthma Consensus [36]. Results are shown in Fig. 3. We found that 100% of patients responded to benralizumab treatment, and 79.6% had a very good response (controlled asthma or complete response), while only nine patients showed a partial response with eight remaining corticosteroid-dependent (although with a reduction in OCS ≥ 50%) and one, who was corticosteroid-dependent and despite managing to discontinue permanently OCS, required two courses of OCS during that year, although a > 50% reduction in OCS use was observed. Of these nine patients with a partial response, six had had their asthma previously treated with a biological agent. No patients were classified as non-responders.

Among the side effects experienced by nine patients (20.5%), the most common ones were arthralgias, headaches, and dysthermia. However, all side effects were mild and there were no treatment discontinuations due to side effects.

### Direct healthcare costs

Table 5 compares the cost of healthcare resources used in the preceding year and in the year with benralizumab therapy. Costs increased during the year following benralizumab treatment initiation due to the price of the biological treatment, but the costs of complementary tests, emergency care and admissions, and oral and inhaled corticosteroids decreased.

**Table 5:** Cost of healthcare resources used

Resources	Cost per patient, mean (SD)		p (Wilcoxon test)
	Previous 12 months (baseline)	At 12 months on benralizumab	
Diagnosis and complementary tests	962.71 (44.87)	218.62 (43.84)	**< 0.001**
Corticosteroids			
Oral	60.79 (32.97)	4.54 (9.40)	**< 0.001**
Inhaled	183.44 (89.62)	105.62 (50.23)	**< 0.001**
Emergency Department visits	1585.94 (1003.78)	267.29 (596.48)	**< 0.001**
Admissions	1599.33 (2937.06)	615.13 (2601.51)	**0.003**
Biological treatment for asthma	7151.63 (7778)	12,832.04 (2020.17)	**< 0.001**
Total, cost per patient	11,544 (9137)	14,043 (3822)	**0.006**

At baseline and at 12-months of benralizumab treatment

### Cost-effectiveness analysis (CEA)

#### Decrease in the number of exacerbations

Table 6 shows the results of the CEA based on the reduction of severe exacerbations. As shown, an incremental cost of €602/year is required to avoid one severe exacerbation, and €3300/year to avoid any exacerbation in a given patient. On the other hand, the number of severe exacerbations correlates (Pearson’s R coefficient) with the cost of OCS (R = 0.839; 95% CI 0.709–0.913) and with the cost of emergency department visits (R = 0.849; 95% CI 0.726–0.919).

**Table 6:** Cost-effectiveness analysis of benralizumab treatment based on severe exacerbations

Mean (SD) per patient	Previous 12 months (baseline)	At 12 months on benralizumab
No. of exacerbations	5.50 (2.63)	0.66 (0.94)
Direct healthcare costs (in €)	10,292 (7885)	13,204 (2145)
Difference in		
% reduction of exacerbations	88.14	
Costs (€)	2912	
Total cost to reduce 1 exacerbation (€)	602	
p value	< 0.001	
Cost-effectiveness ratio	3304	
Cohen’s d	2.00 (1.49–2.51)	

**Fig. 4:**
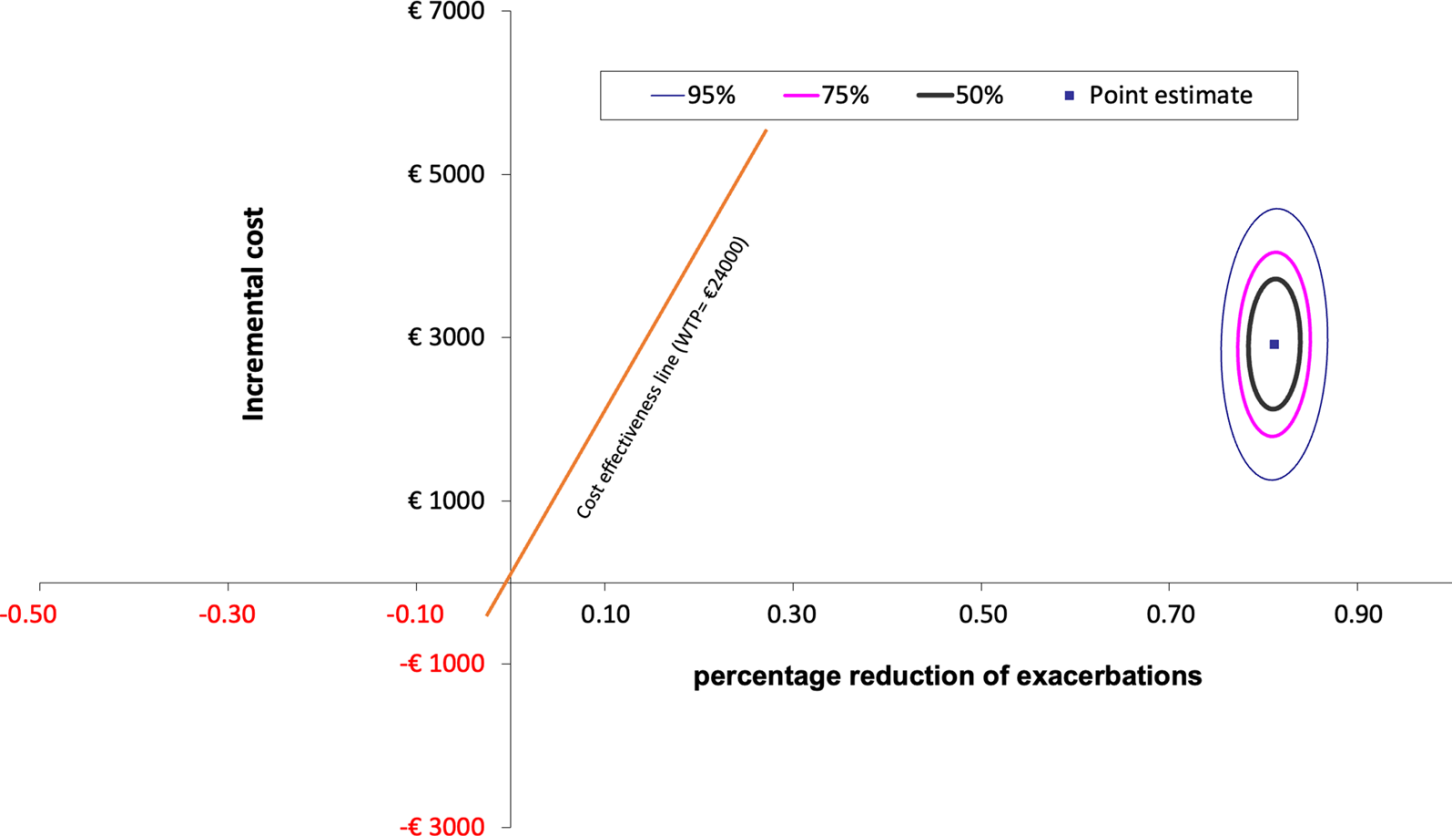
Cost-effectiveness plane for benralizumab versus baseline treatment periods. Confidence ellipses using bootstrap statistics

The Fig. 4 shows the incremental cost-effectiveness of the new treatment with benralizumab compared with the previously used treatment option (baseline situation) in patients with refractory eosinophilic asthma. Values shown indicate the uncertainty surrounding the incremental cost-effectiveness ratio. Each blue dot thus represents a patient (one cost, one effectiveness). The Monte Carlo simulation extrapolates the results to a hypothetical cohort of 1000 patients. Any point below the orange line (patient with its cost and effectiveness) indicates that the procedure is efficient in our country.

#### Asthma control test

Table 7 shows the results of the estimated cost (in Euros/year) to achieve a 1-point ACT increase and a 3-point increase, and to achieve an ACT score > 20 (starting from an ACT score of 13 points, the baseline mean). It also provides a cost-effectiveness analysis expressed as a function of the percentage of effectiveness achieved by the increase of the ACT score.

**Table 7:** Cost-effectiveness analysis (ACT score) of benralizumab treatment

Period	Mean (SD) per patient			Increase		
	Direct healthcare costs (in €)	Asthma control test		Costs	ACT score	% effectiveness
		Score	Effectiveness rate			
Previous 12 months (baseline)	11,544 (9137)	13.71 (4.01)	0.549 (0.151)	2499	7.62	0.305
At 12 months on benralizumab	14,043 (3822)	21.33 (2.21)	0.853 (0.087)			
Cost/effectiveness (per point gained in the ACT)				327.95		
Cost of a 3-point increase in the ACT [32]				983.86		
Cost of a 7-point increase (from 13 to 20 in the ACT)				2295.67		
Cost-effectiveness (% increase in the ACT score)				8201		

**Fig. 5:**
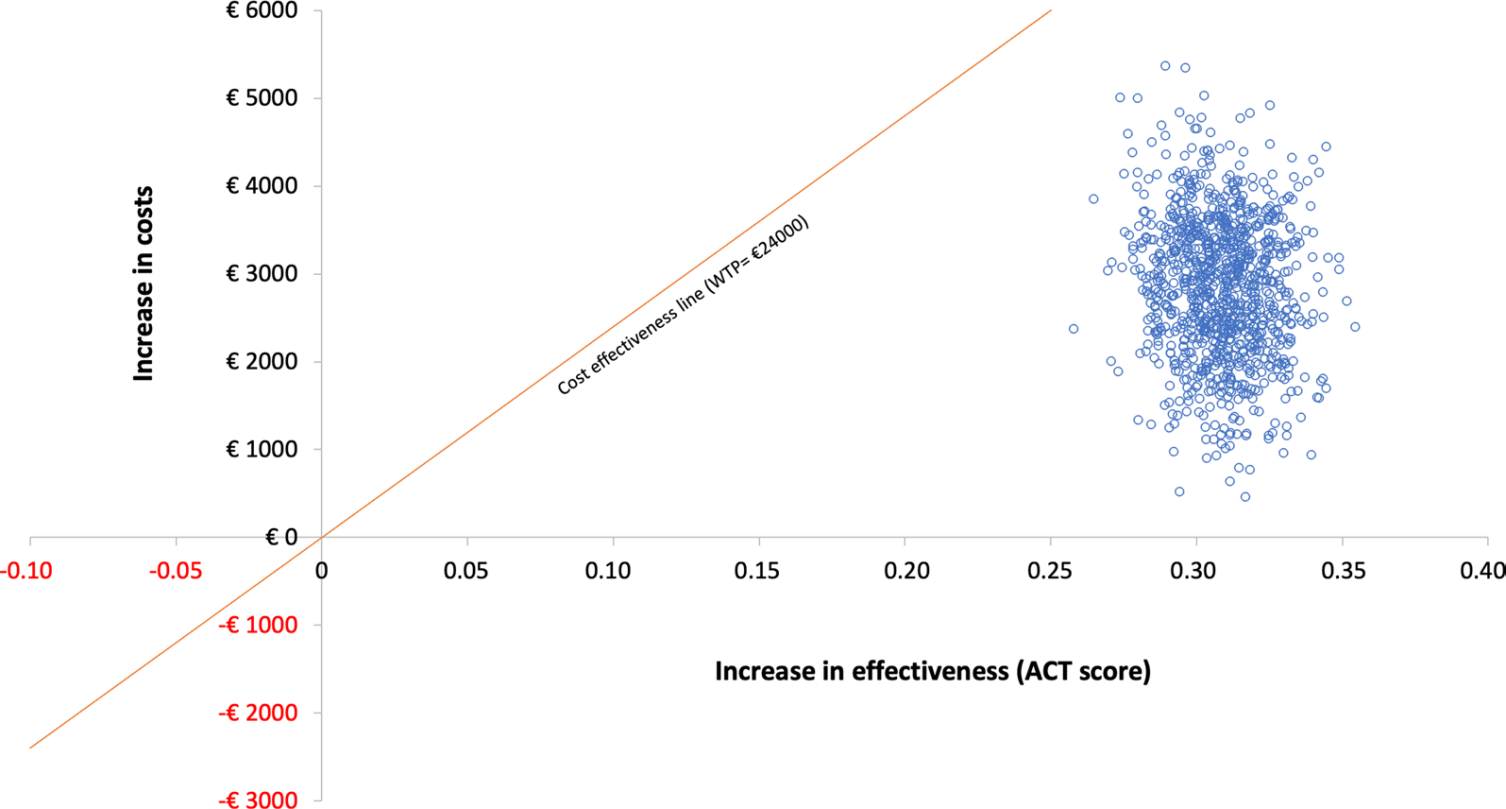
Cost-effectiveness plane (based on the ACT scores) for benralizumab versus baseline treatment periods. Monte Carlo simulation

Figure 5 shows the incremental cost-effectiveness for benralizumab versus baseline 1-year treatment periods, using a Monte Carlo simulation of 10,000 patients.

### Cost-utility analysis (CUA)

The utility gained at 1 year of benralizumab treatment versus the baseline treatment period was 0.138 QALYs (incremental utility) while the incremental cost was €2499. The Table 8 shows the cost-utility analysis carried out. The incremental cost-utility ratio (ICUR) obtained was €18,177/QALY.

**Table 8:** Cost-utility analysis of benralizumab at 1 year of treatment

Period	Mean utility (SD)	Mean total cost (Euros)	Increase		Incremental cost utility ratio
			Utility	Costs	
Previous 12 months (baseline)	0.729 (0.071)	11,544 (9137)	0.138	2499	18,177
At 12 months on benralizumab	0.867 (0.039)	14,043 (3822)			

**Fig. 6:**
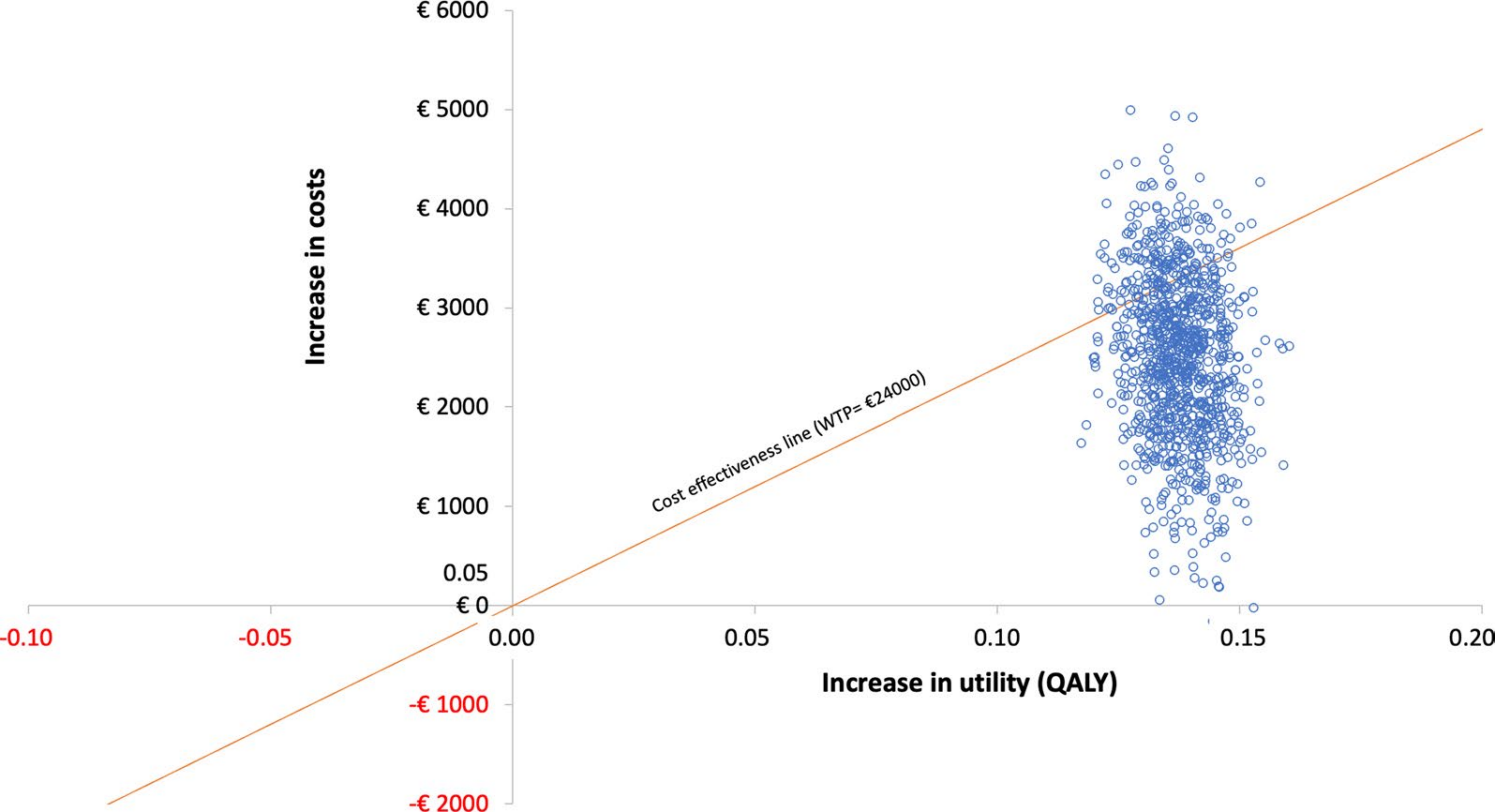
Cost-utility plane (QALY gain) for benralizumab versus baseline treatment periods. Monte Carlo simulation

Figure 6 shows the incremental cost-utility of benralizumab versus baseline treatment periods (other biological treatments) at 1 year of treatment, using a Monte Carlo simulation of 10,000 patients. Values shown indicate the uncertainty surrounding the ICUR. For a WTP of €24,000, the likelihood of benralizumab providing a better cost-utility compared with the baseline treatment option was 80.9%.

### Net benefit

The Net Monetary Benefit (NMB) obtained with benralizumab was €813 (NMB = [0.138 * 24,000] − 2499 = 813) which means that, as it is higher than 0, it is an efficient treatment option compared to the baseline option.

The positive NHB outcome suggests that it is a measure that improves the general health of the population (NHB = 0.138 − [2499/24,000] = 0.034).

## Discussion

This was an effectiveness and pharmacoeconomics study on the use of benralizumab in real life in patients with refractory eosinophilic asthma.

Before prescribing a biological product, essential aspects such as a correct diagnosis, adherence to the prescribed therapeutic plan, and appropriate inhaler technique (if applicable) should be reviewed. Once they have been confirmed, therapy with biologics should be considered in case of a lack of clinical effectiveness [1]. In recent years, the health authorities have approved four new drugs classified as biologics, in addition to omalizumab: mepolizumab, benralizumab, reslizumab, and more recently dupilumab (although marketing approval for the latter has not been granted in Spain yet). However, little is known about their effectiveness in actual clinical practice, and even less is known about their cost-effectiveness in usual care settings. The main objective of the present study was to address these questions.

With regard to the effectiveness of a 1-year benralizumab treatment in real life, our findings have shown a significant reduction in the number of emergency department visits, severe exacerbations, and use of OCS as well as an improvement in asthma control and lung function, in line with the SIROCCO [19], CALIMA [20], and ZONDA [21] pivotal studies. Likewise, real-life data showing similar results after 6 months [23, 45] and 1 year of treatment [24, 25, 46] have been published. Our study revealed that 65.9% of patients had required zero emergency department visits during the 1-year treatment with benralizumab, a slightly better outcome than the 40% found by Kavanagh et al. [24], while a 55.6% reduction in the number of corticosteroid dependent patients was obtained, a value similar to the 51.4% described by these authors. We also observed a significant decrease in the number of OCS courses used. Recent studies have shown that OCS courses are associated with a greater probability of experiencing side effects [47] and therefore a reduction in the use of OCS may improve outcomes in patients with asthma.

Another interesting finding was the reduction in ICS which we had already observed in our previous real-life study with benralizumab during 6 months [23] and which has also been demonstrated in other real-life studies [24]. This, however, had not been shown in pivotal studies, and the randomized controlled SHAMAL study is currently under way to assess if the use of ICS may be decreased in these patients (ClinicalTrials.gov Identifier: NCT04159519).

The percentage of responders in our investigation (100%) was again higher than in Kavanagh et al.’s study [24] where 13.8% of patients did not respond. A universally accepted consensus to define a complete response or super response is lacking. The response criteria used in our study were those recommended by the Spanish Consensus on Severe Asthma [36], which are more restrictive than those described by Kavanagh et al. [24] (reduction of ≥ 50% in annualised exacerbation rate or in maintenance OCS dose after 48 weeks of treatment) as they include parameters such as asthma control and lung function. Thus, in our case, a patient with a complete response was defined as a patient with zero exacerbations during 1 year, an ACT score ≥ 20, an FEV_1_ ≥ 80%, and no maintenance OCS. Based on these criteria, only 12 patients (27.3%) showed a complete response. Kavanagh et al. [24] defined super responders as patients with zero exacerbations and no maintenance OCS for asthma, and 39% of their patients met these criteria. With these criteria, 59.1% of our patients would qualify as super responders. Our findings are better than those obtained in the pivotal studies SIROCCO [19], CALIMA [20], and ZONDA [21] and in certain real-life studies conducted [24], probably because of the significant eosinophilic inflammation experienced by our patients who had a mean blood eosinophil count of 718.3 ± 287.5 cells/μL.

With regard to safety, no serious side effects were found and no treatment was discontinued due to side effects. These results reinforce the data provided by the pivotal studies on benralizumab safety which showed low rates of discontinuation due to side effects (2% in SIROCCO [19] and CALIMA [20], and 2–3% in BORA [22]).

### Pharmacoeconomics analysis

All the pharmacoeconomic parameters analysed show that treatment with benralizumab is a cost-effective option as an add-on therapy in patients with refractory eosinophilic asthma.

The total cost per patient during the preceding year was €11,544 whereas, during the subsequent year on treatment with benralizumab, this was €14,043. This annual increase of €2499 was the result of the price of the biological treatment and the decrease in costs related to hospital admissions, emergency department visits, and OCS or ICS use. To date, there has only been one study published analysing benralizumab cost-effectiveness based on pivotal studies [48], in which a total increase per patient and year was also found and was also the result of the increase in costs from the use of benralizumab. This has also been evidenced with other biological treatments for asthma as shown in several systematic reviews [49, 50] demonstrating that time horizon and drug price are among the key drivers of the incremental cost-effectiveness ratio.

Incremental costs of €602/year and €3300/year to avoid a severe exacerbation and any exacerbation, respectively, in a given patient were found. Furthermore, for a 1-point ACT score increase, an incremental cost of €327.95/year was found, and for a 3-point increase the incremental cost was €983.86/year. These costs are significantly lower compared with those found in other pharmacoeconomics studies for other asthma biologics such as omalizumab [11, 51] and mepolizumab [15]. This is most likely due to the fact that current prices are lower, to differences in healthcare systems, and to a greater effectiveness of benralizumab in our study resulting from the fact that patients were managed in specialised asthma units, which enabled us to apply strict inclusion criteria and enrol patients with very severe asthma and severe eosinophilic inflammation.

With effectiveness measured as a percentage of gain in ACT score and taking into account that an efficient alternative in Spain is considered when the cost-effectiveness threshold lies between €22,000 and €24,000 [52], the probability of benralizumab being a cost-effective option for a WTP of €24,000 was 99.8%, and it was found to be dominant (more effective and less costly) in almost 10% compared with the baseline alternative.

The NMB obtained was positive, indicating that benralizumab was a more efficient alternative than its comparator. Furthermore, the positive incremental NMB indicates that the intervention was cost-effective compared to the alternative regimen based on the WTP threshold in Spain (€22,000–€24,000). Thus, the cost of obtaining an additional benefit was below the maximum amount decision makers would be willing to pay for this benefit. Furthermore, the positive NHB outcome suggests that it is a measure that improves the general health of the population.

In addition, at a WTP threshold of €24,000, the probability of benralizumab having a better cost-utility for a WTP of €24,000 was 81% and was found to be dominant (more utilities and lower costs) in 8.3% compared with the baseline alternative.

Overall, based on our study, benralizumab in association with the standard treatment of refractory eosinophilic asthma appears to be a cost-effective option in Spain with a cost < €24,000/QALY gained, whereas in a prior cost-effectiveness study in Sweden based on pivotal studies [48] this cost ranged between €40,000 and €70,000/QALY gained. These considerably higher values are most likely due to differences in prices between the two countries and differences between both national healthcare systems. In fact, cost-effectiveness results for a particular drug may vary from country to country because of these differences. Thus, mepolizumab was reported to be cost-effective for the treatment of severe eosinophilic asthma in the United Kingdom [53] but was found not to be cost-effective for similar patients in the United States at commonly cited WTP thresholds [15].

### Limitations

Our study has some limitations. Because this was a real-life study, there was no placebo control group and therefore a placebo effect could not be assessed. Additionally, as a result of the pandemic caused by SARS-CoV-2, spirometry at 1 year of treatment with benralizumab could not be performed in four of 44 patients. This explains why figures in Tables 1 and 2 are different. Likewise, FeNO measurements could not be assessed in these patients and were excluded from the analysis.

Its strengths, on the other hand, lie in the fact that, to our knowledge, this is the only study of benralizumab with real life data to date. In addition, it is a multicentre study that was conducted at two different severe asthma units with a broad experience in the management and treatment of this disease. Finally, this was an independent study with no external funding and without the involvement of any pharmaceutical company.

## Conclusions

We observed a clear improvement in asthma control and lung function as well as a reduction in severe exacerbations, emergency department visits, use of OCS (both as corticosteroid courses and maintenance therapy), and ICS in patients with refractory eosinophilic asthma treated with benralizumab for 1 year. Moreover, benralizumab appears to be a cost-effective option (less than €24,000/QALY) as an add-on treatment in patients with refractory eosinophilic asthma in Spain.

## Data Availability

The data sets analysed during the current study are available from the corresponding author upon reasonable request.

## Abbreviations

ACT: Asthma control test
AERD: Aspirin-exacerbated respiratory disease
BD: Bronchodilator
BMI: Body mass index
FeNO: Fractional exhaled nitric oxide
FEV1: Forced expiratory volume in 1 s
GINA: Global Initiative for Asthma
ICER: Incremental cost-effectiveness ratio
ICS: Inhaled corticosteroids
IL-5: Interleukin-5
IL-5R: Interleukin-5 receptor
IR: Interquartile range
LABA: Long-acting β2-agonists
OCS: Oral corticosteroids
